# Improved genome assembly and evidence-based global gene model set for the chordate *Ciona intestinalis*: new insight into intron and operon populations

**DOI:** 10.1186/gb-2008-9-10-r152

**Published:** 2008-10-14

**Authors:** Yutaka Satou, Katsuhiko Mineta, Michio Ogasawara, Yasunori Sasakura, Eiichi Shoguchi, Keisuke Ueno, Lixy Yamada, Jun Matsumoto, Jessica Wasserscheid, Ken Dewar, Graham B Wiley, Simone L Macmil, Bruce A Roe, Robert W Zeller, Kenneth EM Hastings, Patrick Lemaire, Erika Lindquist, Toshinori Endo, Kohji Hotta, Kazuo Inaba

**Affiliations:** 1 Department of Zoology, Graduate School of Science, Kyoto University, Sakyo, Kyoto, 606-8502, Japan; 2 Graduate School of Information Science and Technology, Hokkaido University, N14W9, Sapporo, 060-0814, Japan; 3 Graduate School of Science and Technology, Chiba University, Inage, Chiba, 263-8522, Japan; 4 Shimoda Marine Research Center, University of Tsukuba, Shimoda, Shizuoka, 415-0025, Japan; 5 Division of Disease Proteomics, Institute for Enzyme Research, The University of Tokushima, 3-15-18 Kuramoto-cho, Tokushima, 770-8503, Japan; 6 Montreal Neurological Institute and Departments of Neurology and Neurosurgery and Biology, McGill University, 3801 University St, Montreal, Quebec, H3A 2B4, Canada; 7 McGill University and Genome Quebec Innovation Centre, and Department of Human Genetics, McGill University, Montreal, Quebec, H3A 2B4, Canada; 8 Advanced Center for Genome Technology, and Department of Chemistry and Biochemistry, University of Oklahoma, Norman, Oklahoma, 73019-0370, USA; 9 Department of Biology, San Diego State University, San Diego, California, 92182-4614, USA; 10 Institut de Biologie du Developpement de Marseille Luminy (IBDML), CNRS-UMR6216/Universite de la Mediterranee Aix-Marseille, Marseille, 13288, France; 11 DOE Joint Genome Institute, Genomic Technologies Department, 2800 Mitchell Drive, Walnut Creek, California, 94598, USA; 12 Faculty of Science and Technology, Keio University, Kouhoku, Yokohama, 223-8522, Japan

## Abstract

**Background:**

The draft genome sequence of the ascidian *Ciona intestinalis*, along with associated gene models, has been a valuable research resource. However, recently accumulated expressed sequence tag (EST)/cDNA data have revealed numerous inconsistencies with the gene models due in part to intrinsic limitations in gene prediction programs and in part to the fragmented nature of the assembly.

**Results:**

We have prepared a less-fragmented assembly on the basis of scaffold-joining guided by paired-end EST and bacterial artificial chromosome (BAC) sequences, and BAC chromosomal *in situ *hybridization data. The new assembly (115.2 Mb) is similar in length to the initial assembly (116.7 Mb) but contains 1,272 (approximately 50%) fewer scaffolds. The largest scaffold in the new assembly incorporates 95 initial-assembly scaffolds. In conjunction with the new assembly, we have prepared a greatly improved global gene model set strictly correlated with the extensive currently available EST data. The total gene number (15,254) is similar to that of the initial set (15,582), but the new set includes 3,330 models at genomic sites where none were present in the initial set, and 1,779 models that represent fusions of multiple previously incomplete models. In approximately half, 5'-ends were precisely mapped using 5'-full-length ESTs, an important refinement even in otherwise unchanged models.

**Conclusion:**

Using these new resources, we identify a population of non-canonical (non-GT-AG) introns and also find that approximately 20% of *Ciona *genes reside in operons and that operons contain a high proportion of single-exon genes. Thus, the present dataset provides an opportunity to analyze the *Ciona *genome much more precisely than ever.

## Abstract 2

An improved assembly of the Ciona intestinalis genome reveals that it contains non-canonical introns and that about 20% of Ciona genes reside in operons.

## Background

The tunicates are a chordate sister group of the vertebrates that has long been of great interest to evolutionary and developmental biologists. Vertebrates and tunicates have genomic similarities, reflecting their evolutionary relationship, and also differences. Differences of particular interest include the much smaller genome of tunicates [1] and the occurrence in tunicates, but not vertebrates, of spliced leader (SL) pre-mRNA trans-splicing (SL trans-splicing) and its use, in part, to generate individual mRNAs from polycistronic transcription units, or operons [2-4].

The ascidian *Ciona intestinalis *is perhaps the best-characterized tunicate. The version 1 *Ciona *draft genome sequence and assembly was published in December 2002 [1] and a major assembly update (version 2) was released in March 2005 [5]. Several annotations based on assembly versions 1 and 2 have been published [1,6,7], but the gene model predictions have not been systematically evaluated and, in practice, are often found to be inconsistent with the growing body of experimental cDNA-based sequence data. Since the initial publication of the draft genome, a wide variety and great depth of data useful for gene annotation has been accumulated, whose large-scale integration into the annotation process would greatly improve the accuracy of the gene model set.

The most important factor contributing to currently unsatisfactory annotations is probably the intrinsically limited accuracy of gene prediction programs. Such predictions are imperfect even for uncomplicated loci, but particular difficulties are encountered in the case of unusual structures such as *Ciona *operons, which contain two or more genes directly abutted without intergenic regions [2]. Universal pipelines for genome annotations generally fail to correctly predict such unusual structures; two or more distinct genes within an operon are often wrongly predicted as artifactually fused single genes. Since a significant fraction of the total *Ciona *gene number is encoded in operons, such mis-annotations can cause serious errors genome-wide.

Another factor contributing to incorrect gene models is the significant residual fragmentation of the genome sequence assemblies. In many cases, 5' and 3' sequence reads from individual expressed sequence tag (EST) clones or full-insert sequences of cDNA clones map to different gene models on separate scaffolds. Hundreds of loci are affected by such artifactual splitting of gene models.

Taking advantage of the great breadth and depth of published and as yet unpublished mRNA-based sequence evidence, including extensive 5'-full-length EST data, and additional bacterial artificial chromosome (BAC)-based end-sequence and chromosomal *in situ *hybridization data, we have generated an updated *Ciona *genome assembly and a new gene model set. The assembly is a marked improvement in terms of residual fragmentation, and the gene model set is far more consistent with the cDNA evidence than existing model sets. The assembly and gene model set together represent an important research resource update for *Ciona *genomic studies. Using these updated resources, we report several novel insights into the *Ciona *genome. We establish the existence of a population of non-GT-AG introns, and show that operons are far more numerous than previously estimated and contain a high proportion of single-exon genes.

## Results and discussion

### Comparison of assembly versions 1 and 2

We first compared the two available assemblies of the *C. intestinalis *draft genome sequence [1], version 1 (December 2002, 116.7 Mb) and version 2 (March 2005, 173 Mb). The version 2 genome has apparently better N50 scaffold sizes (2.6 Mb versus 187 kb) and N50 scaffold number (17 versus 174), while the total number of scaffolds is much larger than in version 1 (4,390 versus 2,501) and the 173 Mb total length is greater than expected for the *Ciona *genome (155 Mb including euchromatic and non-euchromatic regions [8]).

From a total of 1,179,850 available *Ciona *ESTs that were obtained from conventional (that is, oligo(dT)-primed, non-5'-RACE) cDNA libraries, we were able to confidently map 881,492 onto version 1 and a smaller number, 850,361, onto version 2 (the mapping criterion was alignment over >90% of the entire EST length with >95% identity). A significant fraction of ESTs (25% for version 1 and 28% for version 2) failed to be mapped under this stringent mapping condition. However, under less stringent (default) mapping criteria, almost the entire population (96% for version 1 and 92% for version 2) of ESTs was mapped; 1,133,688 and 1,087,716 ESTs were mapped onto version 1 and 2 assemblies, respectively. The failure of 25-28% of ESTs to be mapped at the higher but not the lower stringency criteria presumably reflect EST sequencing errors and/or allelic variation.

The fact that more ESTs were mapped to assembly version 1 suggests that version 1 contains genes missing from version 2 and, in fact, 733 of the 15,582 version 1 models (approximately 5%) could not be mapped onto the version 2 assembly. Examples include well-characterized genes such those encoding a myosin regulatory light chain MRLC5 [DDBJ: AK174195] and troponin I [GenBank: U94693].

The two assemblies also differed in the relative number of unique versus duplicated genes. Of the confidently mapped ESTs, 856,735 (97%) and 744,958 (88%) mapped onto unique locations of the version 1 and 2 genomes, respectively, the remainder mapping to multiple sites with similar alignment scores. This observation indicates that version 2 contains more instances of very closely related genes. Such duplication, which could perhaps include allelic variants, presumably contributes to the greater total length of the version 2 genome. Taken together, these observations suggested that the version 1 assembly was more suitable for global gene annotation.

We have assembled a large dataset (approximately 1.4 million sequences) of mRNA-based sequence evidence, including extensive 5'-full-length EST data (Table 1). Using these data and additional chromosomal *in situ *hybridization and BAC-based end-sequence data [9], we have generated both an updated *Ciona *genome assembly based on version 1, and a new and more accurate gene model set.

**Table 1:** cDNA sequence evidence used in the present study

ESTs (conventional cDNA clones)*	1,179,850
5' EST	589,329
3' EST	590,521
5'-full-length ESTs	202,535
Oligo-capping cDNA library-derived ESTs^†^	2,079
Spliced-leader mRNA derived ESTs^‡^	199,947
5'-RACEs from oligo-capping cDNA pool^§^	509
Full insert cDNA sequences^¶^	8,877

*There were 672,390 ESTs published before [1,12]. The rest of the ESTs were produced recently and high quality reads among them were deposited in the GeneBank database ([GenBank: FF685517-FF836289] and [GenBank:FF848360-FG007279]). ^†^Described in [2]. ^‡^Pooled data from two sets of SL-based reverse-transcription PCR analyses. One dataset consisted of 19,571 sequences derived from oligo(dT)-primed cDNA of mRNA from pooled embryonic/adult stages and several adult tissues (Y Satou *et al*., unpublished data). The other consisted of 180,376 SL-containing sequences >30 nucleotides derived from random-hexamer-primed cDNA of mRNA from tailbud embryos (J Matsumoto et al, manuscript in preparation). ^§^From a study by oligo-capping 5'-RACE for determining 5'-ends of mRNAs encoding transcription factors (Y Satou *et al*., unpublished data). ^¶^Sequences of full-inserts of cDNA clones downloaded from the public database.

### The KH assembly: linkage of version 1 scaffolds

The new assembly, termed the KH assembly for Kyoto Hoya (hoya is a Japanese word for ascidian), was generated from the version 1 assembly by an evidence-based process of scaffold joining, coupled with the removal of small scaffolds that did not appear to contain expressed genes or that appeared to be variant duplicates of regions better represented in other scaffolds (Additional data files 1 and 2).

We observed during our EST mapping analysis 11,516 cases in which the 5' and 3' EST mate-pair sequences derived from a single cDNA clone mapped to different version 1 scaffolds. This finding indicated the occurrence of many instances in which genes had been artifactually split onto two or more version 1 scaffolds. In some cases this resulted from a small within-gene gap in the genome sequence, and in some cases it involved scaffolds that appeared to overlap at their ends but could not be merged by the assembly program because of variation in the two versions of the overlap sequence.

**Figure 1:**
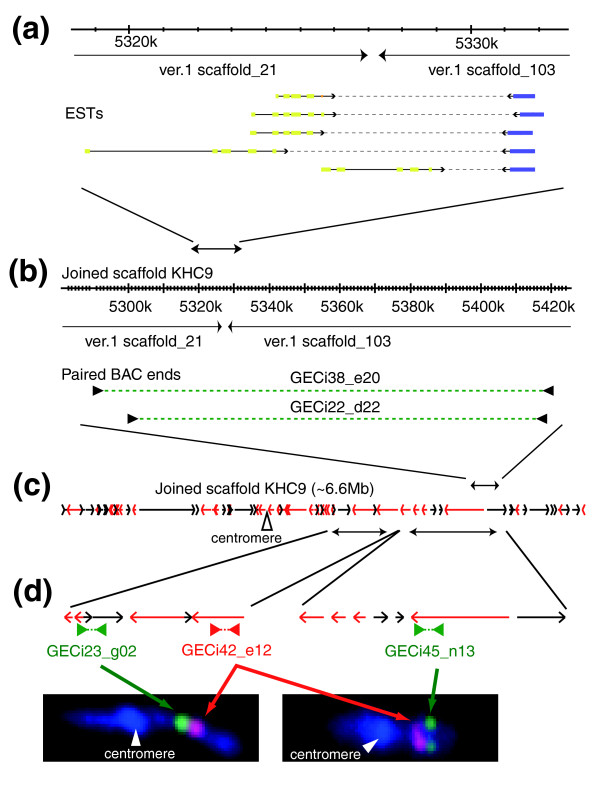
Concordant identification of linkage between version 1 scaffolds from EST mate pairs, and BAC paired-end sequences. **(a) **Multiple 5'- and 3'-EST mate pairs identified a linkage between version 1 scaffolds 21 and 103. **(b) **Paired end sequence data of two independent BAC clones also identified this joined-scaffold linkage. **(c) **Identification of such linkages and FISH data constitute a larger scaffold representing chromosome 9. This new scaffold includes 61 of version 1 scaffolds. Black and red arrows indicate version 1 scaffolds in leftward and rightward directions. **(d) **FISH data are used to orient and place tentative joined scaffolds, which are built by EST mate pairs and paired BAC ends, on chromosomes. Left panel: two-color FISH of GECi23_g02 (green) and GECi42_e12 (red) BAC clones, which are mapped onto the same tentative joined scaffold, determines the orientation of this tentative joined-scaffold on the chromosome 9. Right panel: similarly, two-color FISH of GECi45_n13 (green) and GECi42_e12 (red) BAC clones, which are mapped onto different tentative joined-scaffolds, indicates that these two tentative joined scaffolds are in this order on chromosome 9. White arrowheads indicate the centromere.

**Figure 2:**
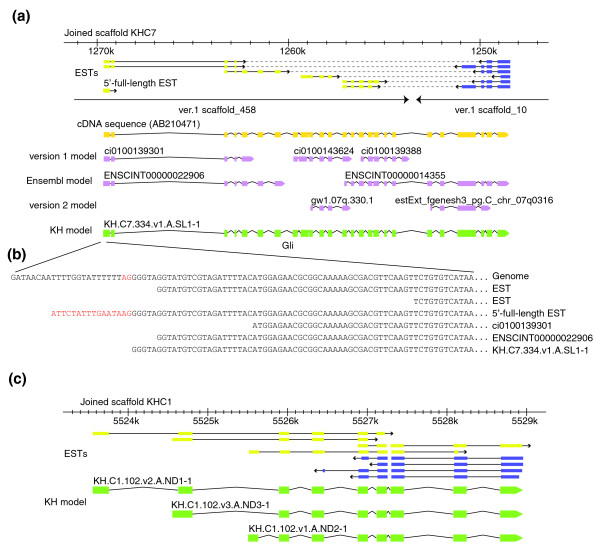
Improvement of gene models. **(a) **Improvement of a gene model for *Gli*, including the joining of two JGI version 1 scaffolds. 5'-ESTs and 3'-ESTs are shown as yellow and purple boxes and EST pairs are connected by dashed lines. Multiple EST pairs indicate that this locus is artifactually split into two version 1 scaffolds. This *Gli *gene locus was not precisely predicted in the previous studies (exons are indicated by pink boxes and joined by lines). The new gene model (green boxes) precisely coincides with the structure of a cDNA sequence (yellow boxes) and ESTs. **(b) **The alignment of ESTs and gene models with the genome sequence around the 5'-end of the *Gli *locus. The 5'-full-length EST shown here has the spliced leader sequence (red letters), which is not aligned with the genome sequence because it is appended to *Gli *mRNA by trans-splicing. The acceptor dinucleotide for this trans-splicing is shown in red in the genome sequence. Note that only the new model precisely represents the 5'-end of this locus. **(c) **A gene locus that had not been modeled in previous annotations. Although 5'-ESTs (yellow boxes) and 3'-ESTs (purple boxes) indicate the existence of genes in this region, no previous model sets have included models in this region. Two gene models for this locus were built on the basis of EST evidence.

We used 5' and 3' EST mate-pair sequences to link version 1 scaffolds into 'joined scaffolds' in the KH assembly. To eliminate possible artifacts due to rare chimeric cDNA clones resulting from ligation of two independent cDNA molecules into a single clone, we joined scaffolds only when multiple independent EST pairs indicated the same linkages, and these ESTs mapped to sites within 5 kb of scaffold ends or internal scaffold sequence gaps (see Figures 1a and 2a for examples). Where version 1 scaffolds were joined across a within-gene gap, the joint was marked in the KH genome sequence by a run of Ns (see Materials and methods). In total, 727 linkages were generated on the basis of EST mate-pair sequence data.

Additional joined-scaffolds were established on the basis of a set of 8,875 BAC paired-end sequences [1,10], and chromosome mapping fluorescent *in situ *hybridization (FISH) data for more than 170 BACs [9]. As shown in Figure 1, many joined-scaffold linkages were supported both by multiple concordant EST mate-pairs, and by BAC paired-end sequence data, which supports the validity of EST mate-pair-based joining. The scaffold-joining process was efficient and resulted in some long chains; the largest KH joined-scaffold, approximately 10 Mb in length, incorporated 95 version 1 scaffolds. The distribution within the KH assembly of version 1 scaffolds, and the nature of the scaffold-joining evidence, are shown as genome browser tracks [11] on our web site [12,13].

The KH assembly contains a total of 1,272 scaffolds, corresponding to the 2,249 version 1 scaffolds onto which we were able to map ESTs. The new assembly showed a better N50 scaffold size (5.2 Mb) and a better N50 scaffold number (9) than either the version 1 or version 2 assemblies. The largest KH scaffold corresponding to each of the 14 chromosomes of *Ciona *(scaffold lengths 1.8-10 Mb) was named according to the chromosome (see nomenclature in Materials and methods). These 14 'chromosome' scaffolds include 68% of the total assembly. The total length of the KH joined-scaffold assembly is very close to that of the original JGI version 1 assembly (115.2 Mb versus 116.7 Mb). It is slightly smaller because 252 small JGI version 1 scaffolds were omitted because either no ESTs mapped to them or any that did also mapped, with a better score, to another scaffold.

### The KH gene model set

We developed an updated, evidence-based gene model set for the KH assembly (Additional data file 3). We began by mapping previous gene model sets onto the KH assembly, including the original version 1 gene models [1], gene model sets based on the version 2 genome made by JGI [14] and Ensembl (build 41) [6], and models we had previously made by a combination of the Wise2 [15] and grailexp [16] programs on the version 1 genome [12]. In addition, we constructed a new gene model set based on updated EST information using the grailexp program [16] and we mapped full-insert sequences of cDNA clones, which were available in the DDBJ/EMBL/GenBank database, onto the genome, regarding them as gene models. Using the Apollo editor [17], we chose for each transcript the model that was the best fit to the experimental evidence and, where necessary, modified it to complete the agreement, including precise identification of mRNA 5'-ends based on 5'-full-length EST data, when available (in about one-half of the models). We regarded genomic regions where paired ESTs and/or full-insert sequences were mapped as gene loci, even where no computational models existed, and determined the best transcript models for each locus. The final set of models were termed KH models.

As an example of the gene model improvements, a locus encoding a *Gli *transcription factor that was not accurately represented by earlier models, and whose 5'- and 3'-segments were located on separate version 1 scaffolds, is shown in Figure 2a. Our new model, which joins version 1 scaffolds 10 and 458, was based on EST mate-pair sequence data and a previously determined cDNA sequence containing the full open reading frame [18].

Comparative genomic analysis provided further confirmation that the 5' and 3' halves of joint-spanning models, like *Gli*, do in fact correspond to contiguous genomic sequences. There were 218 joint-spanning KH models for which both the 5' and 3' halves showed good alignments with the genome of a closely related species, *Ciona savignyi*; blastn E-value <1E-5), and in the great majority of these cases (203/218 = 93%) both halves gave top-scoring alignments with the same *C. savignyi *scaffold. This observation supports the validity of EST mate-pair-based linkages and of joint-spanning models.

In the case of the *Gli *model, we assessed the annotation by confirming that all of the introns have canonical splice donor (GT) and acceptor (AG) dinucleotides, and we refined the model by modifying the 5'-end to fit a 5'-full-length EST mapped onto this locus (Figure 2b). Similar intron boundary and 5'-end verification operations, where 5'-full-length ESTs existed, were manually performed on all KH gene models.

Figure 2c shows an example of a genomic region in which no gene models had been predicted, although EST data clearly showed that there is a gene locus in this region. For this locus, we created three new alternatively spliced models that fit the EST evidence.

We developed a transcript naming system for the KH models that captures several useful kinds of information (see Materials and methods). All alternative transcripts derived from a single gene locus share the first three name-fields, which facilitates informatic manipulation of data at the level of the gene locus, in addition to the level of the individual transcript models. Additional name-fields identify specific alternative transcripts differing in exon use, or in the precise location of 5'- or 3'-ends.

**Figure 3:**
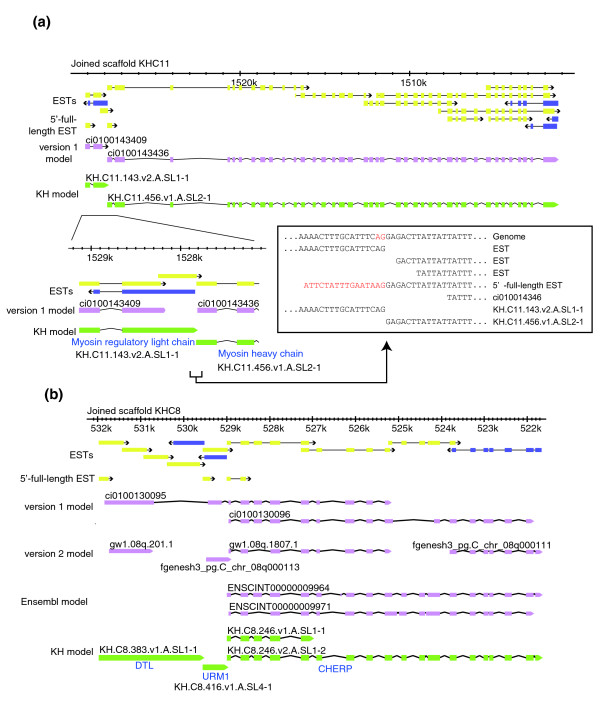
Operons in the *Ciona *genome. In the genomic region indicated, 5'-ESTs (yellow boxes) and 3'-ESTs (purple boxes) clearly indicate that there are **(a) **two and **(b) **three genes encoded. (Note that the genomic region indicated in (a) is not included in the version 2 genome and there are no version 2 gene models.) Previous models (pink boxes) failed to model these loci precisely and the present study yielded gene models that faithfully reflect cDNA evidence. The lower panel in (a) is a magnification of the region around the intergenic region of this operon and the inset shows corresponding DNA sequences.

The great precision of 5'-end determination by 5'-full-length ESTs was a critical input for our gene model work. It provided key data for precisely mapping the 5'-ends of many models, and was particularly important for defining genes in operons. Such improved modeling is shown by the example of an operon containing myosin light chain and myosin heavy chain genes on chromosome 11 (Figure 3a). Reasonably accurate version 1 gene models for these loci existed, but they were incomplete at the 3'-end of the upstream gene and at the 5'-end of the downstream gene. When we refined these ends on the basis of EST data and 5'-full-length ESTs, we found the two genes were precisely abutted, showing the complete absence of intergenic DNA that is a typical feature of *Ciona *operons [2] (and which makes these complex loci difficult for conventional gene prediction programs to interpret.) Another example (Figure 3b) concerns an operon on chromosome 8 that contains three genes homologous to the human genes *DTL*, *URM1 *and *CHERP*. None of the previous gene model sets accurately predicts the structures of all of these operon genes (Figure 3b). Based on EST evidence and precise determination of 5'-ends by 5'-full-length ESTs, we made three precisely abutting gene models here, which again reveal the characteristic organization of *Ciona *operons.

Altogether, the KH gene model set consists of 24,025 transcript models representing 15,254 distinct gene loci (Table 2). This is close to the number of *Ciona *genes estimated by a genomic sequence sampling method, 15,500 ± 3,700 [8]. Among the 24,025 transcript models, 12,615 (corresponding to 7,547 gene loci) had 5'-ends precisely defined by 5'-full-length ESTs, including 11,797 SL trans-spliced transcripts and 818 non-trans-spliced transcripts. Among the remaining 11,410 transcripts for which precise 5'-end definition was not available, we found in-frame stop codons upstream of the longest open reading frames (ORFs) in 7,624 cases. Therefore, the entire protein-coding regions of 20,239 (12,615 + 7,624; 84%) transcripts are expected to be included in the present gene model set.

**Table 2:** Statistics of the KH gene model set

Predicted gene loci	15,254
Predicted transcripts	24,025
Transcripts that putatively encode the full ORF	20,239
Transcript 5'-ends identified by SL ESTs	11,797
Transcript 5'-ends identified by non-SL oligocapping ESTs	818
In-frame stop codons in the 5'-region of the longest ORFs of transcripts not represented by 5'-full-length ESTs	7,624
Operons	1,310
Operon genes	2,909

The total number of KH gene models is close to the number of version 1 gene models (15,852) [1] and the size distributions of exons and introns of the two model sets are similar (data not shown). However, the two model sets are quite distinct. A large number (3,330) of KH loci are located in regions where no version 1 models exist (for example, as in Figure 2c). In addition, 1,779 individual KH loci each incorporate several version 1 models that were partial/incomplete, and 548 version 1 models that incorrectly merged distinct genes are divided in the KH set into separate gene loci. Also, 1,066 KH transcript models (corresponding to 660 gene loci) are built on regions encompassing two (or more) version 1 scaffolds. Finally, many models that were otherwise accurate in the version 1 model set now have, for the first time, precise 5'-end determinations. Thus, the KH model set represents a significant improvement.

### Insight from the KH models: non-canonical (non-GT-AG) introns

The updated assembly and gene model set permit new insight into global features of the *Ciona *genome, including the nature of the intron population. For each KH model, exon-intron boundaries were inspected manually by examination of EST/genome alignments. For most of the 113,879 introns in the KH gene model set (Table 3) the best alignments were consistent with the expected presence of the canonical donor (GT) and acceptor (AG) site dinucleotides. However, for 596 introns the best alignments were not consistent with usage of the GT-AG dinucleotides but were consistent with the use of the known non-canonical dinucleotides GC-AG (556 introns) and AT-AC (40 introns).

**Table 3:** Introns with GT-AG, GC-AG and AT-AC terminal dinucleotides

Terminal dinucleotides	Number of introns
GT-AG	112,989
GC-AG	556
AT-AC	40
Uncategorized*	294

Total	113,879

*The terminal dinucleotides of these introns contain 'N'.

Most eukaryotes contain two distinct types of spliceosomes, which contain either U2 or U12 snRNAs [19]. The vast majority of introns are spliced by U2 spliceosomes and have canonical GT-AG (or rarely GC-AG) terminal dinucleotides. A small minority are spliced by U12 spliceosomes and have non-canonical AT-AC terminal dinucleotides, although a small subset of GT-AG introns are also U12 spliceosome substrates. The present study provides solid evidence that the ascidian genome contains at least 40 AT-AC introns, a set that partly overlaps with those recently predicted computationally [20]. Although the U12 spliceosomal system is widespread among the metazoa, it appears to be absent from the nematodes, almost certainly due to loss during nematode evolution [21]. It is of interest that nematodes, unlike some major metazoan groups, carry out SL trans-splicing [22,23]. The presence of SL trans-splicing coupled with the absence of U12 *cis*-splicing in the nematodes is intriguing, but our results with the trans-splicing organism *Ciona *indicate that SL trans-splicing is compatible with preservation of U12 *cis*-splicing.

### Insight from the KH models: operons

Based on analysis of the JGI version 1 assembly and annotations, we previously estimated that the *Ciona *genome contains 350-450 operons, most of which contain two genes [2]. Because the KH gene model set contains more-complete mRNA 5'-ends than previous model sets, and this is a key criterion for the informatic identification of operons, we also identified candidate operons in the KH assembly and model set. As in our previous study, we operationally defined operons as same-strand gene pairs whose intergenic region was less than 100 base pairs. Application of this search strategy using the KH assembly and models in fact identified 1,310 candidate operons, more than 3-fold more than our previous estimate. 5'-Full-length EST data were available for the great majority of candidate operons, and indicated that upstream and downstream genes were directly abutted without any intergenic DNA, in the pattern previously described [2]. Most candidate operons contained two genes; the largest contained six (Table 4). The total number of genes in candidate operons was 2,909, which represents approximately one-fifth of the total number of genes in the genome. This new, much higher estimate indicates that the operon fraction of the *Ciona *genome is similar to that of *Caenorhabditis *(approximately 15%) [24]. Consistent with the hypothesis that polycistronic pre-RNAs derived from operons are resolved into monocistronic mRNAs by SL trans-splicing [2], we found a very high proportion (1,158 out of 1,599, or 72%) of operon downstream genes were represented by 5'-full-length ESTs.

**Table 4:** Numbers of genes per operon

Number of genes per operon	Number of operons
2	1,079
3	185
4	36
5	8
6	2

**Figure 4:**
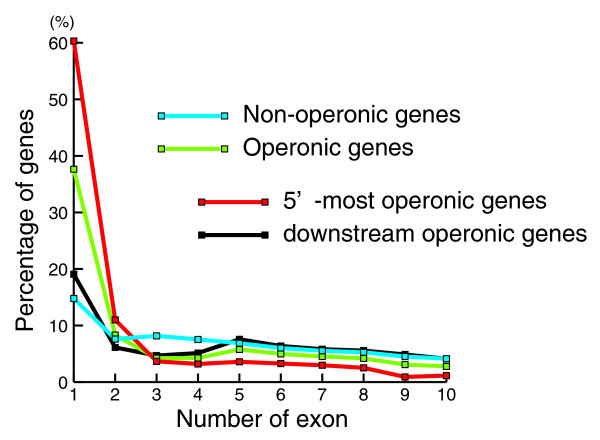
Prevalence of single-exon 5'-most genes in *Ciona *operons. Ratio of genes containing a given number of exons within non-operonic (blue) and operonic (green) gene populations. Red and black lines indicate the ratio within the 5'-most upstream genes encoded in operons and the downstream operonic genes, respectively. Genes with 11 or more exons are not shown in this graph for simplicity. Note that single-exon genes are more prevalent in operons than in the non-operon (monocistronic) gene population, and are especially prevalent among the 5'-most genes of operons.

Operons generate a total of 4,248 distinct mRNAs, with an average length of 1,789 bases. The average length of the 19,777 non-operon (monocistronic) KH mRNAs is 1,893 bases. Despite the similar mRNA lengths, there is a significant difference in exon numbers for operon genes (6.2 exons) and non-operon genes (8.8 exons). The lower average exon number reflects, in part, the presence of a high proportion of single-exon genes in operons (38% versus 15% in non-operon genes). Moreover, single-exon genes are especially over-represented in the 5'-most genes of operons, where they formed the majority (in 790 (60%) of the operons; Figure 4). These single-exon 5'-most genes appear to be *bona fide *protein-coding genes, as opposed to outrons discarded during trans-splicing. They were represented among oligo(dT)-primed cDNA ESTs (and hence they presumably generate polyadenylated transcripts) and many encode protein sequences homologous to those known in other organisms (Figure 3b). The biological significance of the prevalence of single-exon 5'-most genes in *Ciona *operons is not clear, but is likely related to the evolution, function, or gene expression mechanisms of these unusual genetic entities.

## Conclusion

We generated a new reference sequence from the original genome assembly and a new manually curated gene model set, which together represent a significant resource update for *Ciona *genomics studies. The present model set is primarily based on cDNA evidence. The existing *Ciona *cDNA evidence is deep (>10^6 ^sequences) and broad, including samples of a variety of whole-animal developmental stages (eggs to adult), and a variety of individual adult tissues. However, it is still possible that a minor fraction of genes, such as genes expressed only under particular environmental conditions, are not covered by these ESTs. A fraction of previous models not supported by paired ESTs were excluded from the KH model set. A part of them may be real genes or unannotated fragments of genes represented by the KH models, because the encoded protein shows sequence similarity to proteins known in other species (approximately 1,641 loci with <1E-05 blast hits in the human proteome), These are provided as a supplemental model set (see Materials and methods) along with other unsupported or incompletely supported models. In addition, it is probable that a minority of additional genes reside within gaps in the current assembly. This is presumably the case for the small minority of version 2-based gene models that do not map to the KH assembly (48 EST-supported loci). Among the conventional ESTs, 47,511 ESTs (4%) were not mapped anywhere in the KH assembly by the blat program [25] with default parameters. At least a part of these unmapped ESTs may represent *Ciona *genes not included in the KH assembly. Nonetheless, the KH gene set is expected to include the great majority of *Ciona *genes expressed during the normal life cycle. Moreover, we estimate that at least 84% of the KH transcript models contain the complete protein-coding ORF, so the updated resources offer near-complete proteome coverage.

In the present work we exploited EST information to identify linkages between genomic scaffolds. Although these linkages still await refinement through additional genomic DNA sequencing around the joint regions, the existing data are critically useful for gene annotations. In the past decade, whole-genome shotgun technology has generated many draft genome sequences of a variety of different organisms. In many cases, insufficient length of assembled sequences reduces quality of gene annotation, and the approach we have taken in the present study can also be of use for such genomes.

## Materials and methods

### The KH genome assembly

Conventional and 5'-full-length ESTs and full-insert cDNA sequences (Table 1) were mapped onto the JGI version 1 genome assembly by blat [25]. Version 1 scaffolds were joined pair-wise when at least two independent cDNA clones existed whose 5' ESTs mapped to one scaffold and whose 3' ESTs mapped to the other. In most cases EST-based joining linked scaffolds at the ends, although there were several cases in which the EST data clearly indicated that one, or several, version 1 scaffolds mapped to a gap within another version 1 scaffold. These compound, within-scaffold joints were assembled on the same principle as simple pair-wise joints, that is, agreement with the EST data. Scaffolds were also joined on the basis of chromosomal BAC mapping data (FISH) and 12,448 BAC paired-end sequences.

Where nonoverlapping version 1 scaffolds were joined on the basis of EST evidence, the joint was marked in the genome FASTA sequence file by insertion of a run of 125 'N's. Where scaffolds were joined, not by ESTs, but on the basis of BAC end-sequences, the joints were marked by runs of 500 'N's. Some joints within the Cx, or chromosome, scaffolds (see below) were determined solely on the basis of BAC-probe FISH data, and were marked by runs of 1,000 'N's. In such cases the chromosomal order of scaffolds was determined by multicolor FISH using two or more BAC probes on different scaffolds, and scaffold orientations were determined by multicolor FISH using two or more BACs within one scaffold, as described [9]. In rare cases only one BAC was examined in a given scaffold, precluding assessment of orientation. In these cases each end of the scaffold was marked by insertion of a run of 50 lower-case 'n's in addition to the 1,000 'N's marking a FISH-based joint.

The largest scaffold representing each of *Ciona*'s 14 chromosomes was named Cx, where x is the chromosome number. Other joined scaffolds, none of which are currently linked to specific chromosomes, were named Lx, where x is a randomly assigned number ranging from 1 to 173 (numbering order does not reflect scaffold lengths). With one exception, the remaining scaffolds, which are unchanged from the JGI version 1 assembly, were named Sx, where x is the original scaffold number (there are 1,084 total Sx scaffolds). One version 1 scaffold (scaffold_1113), representing the mitochondrial genome, was re-named KHM0; this was not annotated or used in the present study, which was limited to the nuclear genome.

Of the 2,501 scaffolds of the JGI version 1 assembly, 252 mostly small scaffolds were not included in the KH assembly either because no ESTs mapped to them, or any EST that did map to them also mapped to another scaffold with a higher score.

The total number of scaffolds in the KH assembly is 1,272. The KH scaffold sequences are available in Additional data files 1 and 2 and in our web site [13]. This web resource also includes a genome browser. This includes tracks showing: the organization of version 1 scaffolds joined in the KH scaffold, with an indication of the data used to join; the KH and other gene models; all EST and 5'-full-length ESTs that map to the genome; and the 1,310 candidate operons.

### Transcript models

To generate a transcript model set based on current cDNA evidence, we used the grail-exp program [16], which is well-suited for *Ciona *gene prediction [12]. After mapping these new transcript models and previous model sets on the KH assembly, we chose and refined the best models, that is, those giving the greatest agreement with the cDNA/EST data, for each individual locus using the Apollo editor [17]. We did not notice any characteristic errors made by gene prediction programs. Special attention was given to gene models that spanned the joints within joined scaffolds. When non-overlapping version 1 scaffolds were joined by spanning ESTs, we included in the transcript model only sequences present in the genome assembly sequence. Thus, if the spanned genome gap included one or more exons present in the spanning ESTs, these exons were excluded from both the genome assembly, and from the final transcript model. In order that such within-transcript gaps did not frameshift EST ORFs, it was occasionally necessary to introduce additional 'N's in the transcript model in the region corresponding to the genome gap. In cases of overlapping but divergent and unmergeable version 1 scaffold end-sequences, we made transcript models by carefully selecting those exons from the directly repeated overlap region that were the best match with the cDNA data, and avoided inappropriate duplication in the models of identical/similar exons repeated in the genomic sequence. In all cases, final models were prepared by taking the existing models that best fit the cDNA evidence and improving the agreement where possible by manual verification/refinement of intron-exon boundaries and precise localization of 5'-ends on the basis of 5'-full-length ESTs, where available. The KH gene model set is available in Additional data file 3.

Curators assigned ranks of confidence to individual models. Models supported by cDNA data throughout all or most of their lengths were assigned to the 'A' rank (83% of models). Models only partially supported by cDNA data and expected to include imprecise exons or to lack exons were assigned to the 'B' rank. Models in which no clear ORF was found or where uncertainty arose from mismatches between genome and cDNA sequence data or from insufficient cDNA data were assigned to the 'C' rank.

We have also preserved, as a supplemental browser track, a set of gene models predicted by the various *ab initio *prediction programs that do not overlap with KH models and for which there was no paired-EST support. These supplemental models are not part of the KH model set. Among this large set of supplemental models (17,248 models representing approximately 11,476 gene loci) probably very few represent real genes. However, a small number (4,193 models representing approximately 1,641 gene loci) may be real genes or unannotated parts of genes represented by the KH models, because they encode a polypeptide similar to human proteins (<1e-5 by blast search against the IPI (international protein index) human proteome, version 3.29 [26]).

### Naming conventions of transcript and gene models

KH transcript model names consist of six fields delimited by dots (for example, KH.C1.1.v1.A.SL1-1). The first field represents the genome assembly version and, therefore, all the models have the same tag: KH stands for Kyoto Hoya. The second name-field represents the scaffold name (see above for explanation of Cx, Lx, and Sx scaffold names). The third name-field represents the serial number for the gene locus within individual scaffolds. The fourth field specifies gene exon-use alternative transcript variants by number (this number is always preceded by the character 'v'). Transcript models sharing the same set of exons, but differing in the precise location of 5'- or 3'-ends are assigned the same variant number. The fifth name field represents ranks of confidence in the model, as described above. The sixth name-field is concerned with the nature of the 5'- and 3'-ends of the models. The subfield preceding a hyphen refers to the evidence identifying the 5'-end: SL means trans-splice acceptor site precisely defined by 5'-full-length ESTs, nonSL means non-trans-spliced mRNA 5'-end precisely determined by 5'-RACE analysis, and ND means 5'-end identified by conventional (non-5'-RACE) cDNA ESTs that are certain to lack at least several residues at the mRNA 5'-end, and whose trans-splicing status is unknown. The number adjoined to the 5'-end code identifies individual alternative 5'-ends within each locus. The subfield following the hyphen refers to the 3'-end and consists of numbers identifying individual alternative 3'-ends within each locus.

## Abbreviations

BAC: bacterial artificial chromosome; EST: expressed sequence tag; FISH: fluorescent *in situ *hybridization; ORF: open reading frames; SL: spliced leader.

## Authors' contributions

YS designed and organized the present work. YS, KM, MO, YS, ES and LY curated gene models. KU and TE customized the curation softwares. JM, JW, KD, GBW, SM, BAR, RWZ and KEMH provided most of 5'-full-length ESTs. PL and EL provided one-third of ESTs used. KH and KI contributed to this work by critical discussion. YS and KEMH wrote the paper.

## Additional data files

The following additional data are available with the online version of this paper. Additional data file 1 contains joined scaffolds for chromosomes 1-10. Additional data file 2 contains joined scaffolds for chromosomes 11-14, and scaffolds that are not assigned to chromosomes. Additional data file 3 contains transcript models.

## Supplementary Material

Additional data file 1 Joined scaffolds: chromosomes 1-10. Click here for file

Additional data file 2 Joined scaffolds: chromosomes 11-14, and scaffolds that are not assigned to chromosomes. Click here for file

Additional data file 3 Transcript models. Click here for file
